# Quantifying Metal Contamination and Potential Uptake by *Phragmites australis* Adans. (Poaceae) Along a Subtropical River System

**DOI:** 10.3390/plants9070846

**Published:** 2020-07-04

**Authors:** Ndivhuwo R. Netshiongolwe, Ross N. Cuthbert, Mokgale M. Maenetje, Lenin D. Chari, Samuel N. Motitsoe, Ryan J. Wasserman, Linton F. Munyai, Tatenda Dalu

**Affiliations:** 1 Aquatic Systems Research Group, Department of Ecology and Resource Management, University of Venda, Thohoyandou 0950, South Africa; robertndivhuwo@webmail.co.za (N.R.N.); merchattemokgale@gmail.com (M.M.M.); munyailinton@gmail.com (L.F.M.); 2 GEOMAR, Helmholtz-Zentrum für Ozeanforschung Kiel, 24105 Kiel, Germany; rossnoelcuthbert@gmail.com; 3 Centre for Biological Control, Department of Zoology and Entomology, Rhodes University, Grahamstown 6140, South Africa; L.chari@ru.ac.za (L.D.C.); S.motitsoe@gmail.com (S.N.M.); 4 Department of Zoology and Entomology, Rhodes University, Grahamstown 6140, South Africa; ryanwas21@gmail.com; 5 Department of Biological Sciences and Biotechnology, Botswana International University of Science and Technology, Palapye Private Bag 16, Botswana

**Keywords:** enrichment factor, translocation factor, Mvudi river, bioconcentration factor, *Phragmites australis*

## Abstract

Metal pollution is pervasive across terrestrial and aquatic ecosystems owing to anthropogenic activities. Sediments can accrue high concentrations of metals and act as secondary sources, and thus may be valuable indicators of metal contamination across spatiotemporal scales. In aquatic systems, the extent of metal pollution may be further mediated by transference among sediments and living organisms, with plant metal contaminants potentially predictive of underlying sediment concentrations. The present study thus quantifies the extent of metal pollutants (Na, K, Ca, Mg, Cu, Zn, Mn, B, Fe) across multiple study sites and seasons (cool-dry, hot-wet, hot-dry) in a subtropical river system. Furthermore, uptake by a key macrophyte species, *Phragmites australis*, was examined and correlated with sediment pollution levels among different plant parts. Overall, sediment pollution load indices differed seasonally, being significantly highest during the cool-dry season irrespective of sampling location, suggesting that periods with reduced water flows can exacerbate metal pollution levels in riverine sediments. Also, metal concentrations were highest in upstream wetland sites, indicating a capacity for metal sink effects in these areas. Overall, macrophytes contained high concentrations of select metals, however composition and concentrations differed across plant parts, with roots containing particularly high concentrations of Fe and B. Correlations between sediment and macrophyte concentrations were mostly non-significant, whilst stem Mn and Fe concentrations correlated significantly negatively and positively to sediment concentrations, respectively. The present study identifies key spatiotemporal differences in multiple metal contaminants in an understudied subtropical aquatic system that align with hydrological regime differences. Whilst macrophytes were not found to be major accumulators, or predictors, of metal contaminants in this study, they may collectively play a central role in concentration regulation in aquatic systems.

## 1. Introduction

Metals are naturally occurring elements in the geosphere and vary in concentration across and within ecosystem types worldwide [1,2]. The distribution of metals in terrestrial and aquatic systems is mediated by abiotic and biotic factors [3,4]. Climate, temperature, water/soil pH, and dissolved oxygen concentrations all determine metal distributions [3,5,6,7], as do biotic factors such as uptake processes in plants and animals [4,8,9]. Over the past decades, anthropogenic activities have altered biogeochemical cycles, increasing metal accumulation within the environment, with implications for environmental health [10,11]. In many areas, anthropogenic metal contaminants have driven environmental concentrations to surpass critical thresholds, resulting in their presence at toxic levels for various organisms [12,13,14,15]. In aquatic and semi-aquatic systems, however, flora might mediate the translocation of metals, reducing their availability for other organisms and thus metal affects ecosystem processes [16,17]. Whilst metal pollution and remediation dynamics have been well established in developed countries, developing countries have yet to comprehensively examine such processes [18,19,20]. Baseline contamination and remediation studies remain rudimentary, particularly for aquatic systems, hindering our understanding of metal pollution dynamics and potential ecological services provided by native plant species.

In aquatic ecosystems, metals may be present in, and transfer among, sediments, water and living organisms [3]. According to Huang et al. [3], sediments in river ecosystems are heterogeneous assemblages of multitudinous sorbent phases, acting as important repositories and sinks for various contaminants. Sediments are regarded as potential secondary sources of metals that can be valuable indicators of contaminants in aquatic ecosystems; they are therefore useful in studies of metal accumulation [7,13]. Metal mobilities within sediments are variable, and different from those of organic pollutants which are removed by natural processes, such as decomposition. Even the transfer of essential metals differs greatly from that of non-essential metals [7]. Hence, improved insights into mechanisms of accumulation and geochemical distributions of metals are extremely important to gauge the extent of metal pollution in aquatic systems [3,12,21]. In turn, this monitoring is crucial when assessing ecological risks and developing pollution control strategies [22,23,24]. 

Macrophytes represent an important group of plants found within the euphotic zone of aquatic systems. These key organisms play a significant role in nutrient cycling and primary production [4,11,25,26,27], and microhabitat, food, and substrate stability provision in aquatic ecosystems [26,28,29,30]. Aquatic macrophytes can also mediate the translocation of metals in waterbodies. For example, submerged macrophytes such as eelgrass *Vallisneria natans* L. (Hydrocharitaceae), water hornwort *Ceratophyllum demersum* L. (Ceratophyllaceae) and fennel-leaved, pondweed *Potamogeton pectinatus* (L.) Boerner (Potamogetonaceae) can absorb zinc (Zn) via extraction from sediments using roots, and from surrounding water via leaves [4]. In this way, submerged macrophytes form part of the Zn biogeochemical cycle, using active and passive absorption processes to collect and transport this metal [4,11]. Sediments (transport medium) are a major source of metals in macrophytes, being the growth controlling media [31,32]. High concentrations of metals affect the physiological and biochemical activities of the roots, stems and leaves [32]. Furthermore, as macrophyte species respond differently to varying metal concentrations in terms of uptake, enrichment of metals can also vary with macrophytes species [5]. Absorbed nutrients and metals in macrophytes are ultimately released back into aquatic ecosystems during plant decomposition [32]. As a result, uptake and release dynamics in association with shifting biomass make for a variable aquatic landscape with regards to metal concentrations.

The aim of this study is to assess spatiotemporal variations in sediment metal concentrations and determine the accumulation and transfer efficiency of metals in roots, stems and leaves of a key species, *Phragmites australis* Adans. (Poaceae), along a subtropical river system in South Africa. We hypothesised that superficial sediments would consist of high concentrations of metals compared to macrophytes during the cool-dry and hot-dry seasons when water flow is low, and that high metal concentrations will be observed in roots of *P. australis* during this time. By examining roots, stems and leaves separately, we additionally sought to deduce whether different plant parts are better predictors of sediment metal concentrations. Since pollution and land degradation are increasing worldwide and having significant impacts on the environment, data on metal spatiotemporal dynamics and bioavailability are still limited. Accordingly, quantitative studies of metal pollution are required to improve environmental strategies and management of aquatic environments. Through examination of metal contamination in both sediments and key plants, this study will provide knowledge on key source-receptor relationships in aquatic ecosystems.

## 2. Results

### 2.1. Basic water Parameters

Water pH was generally slightly acidic, with means ranging between 5.9 and 6.9, with the exception of site M2 (mean 8.9; cool-dry season), M3 (mean 7.2; hot-dry season) and M1 (mean 7.3; hot-wet season) (Table S1). Sites M3–M5 generally had high conductivity and TDS values across all seasons. Water temperatures increased from the cool-dry (mean range 16–18.3 °C) to hot-wet (mean range 23.0–26.2 °C) (Table S1). No significant seasonal differences (ANOVA, *p* > 0.05) were observed for all water parameters with the exception of water temperature (ANOVA, F_2, 42_ = 6.34, *p* < 0.001). Whereas, significant site variation was observed for conductivity (ANOVA, F_2, 42_ = 4.90, *p* = 0.019) and TDS (ANOVA, F_2, 42_ = 3.62, *p* = 0.045).

### 2.2. Sediment

Sodium, Cu, Zn, Mn, B and Fe were generally high during the cool-dry season, P and soluble S were high during the hot-dry season, and K, Ca and Mg high during the hot-wet season (Table 1). During the cool-dry and hot-wet seasons, site M1 generally had high nutrient concentrations (Table 1). Nutrient and metal concentrations did not show consistent patterns across sites and seasons. Phosphorus, K, Ca, Mg, Mn and soluble S were significantly different (ANOVA, *p* < 0.05) across study sites, whereas, P, Na, K, Ca, Mg, Cu, Zn, B, TOC and Fe concentrations were significantly different (ANOVA, *p* < 0.05) across seasons (Table S2). 

### 2.3. Sediment Quality Indices

The enrichment factors were highly varied across the study sites and seasons (Figure S2). During the cool-dry season, K (all sites), Mg (all sites) and B (sites M2, M4 and M5) showed deficiency to minimal enrichment with extreme enrichment for Fe and Mn (Figure S2a). During the hot-dry season, Cu (all sites), Zn (all sites), B (all sites) and K (site M3) were deficiency to minimally enriched, whilst Ca (sites M1–M3) and Mn (sites M2 and M3) were extremely enriched (Figure S2b). Copper (all sites), Zn (all sites), B (all sites) and K (site M2) were deficiency to minimally enriched, whereas Ca (all sites), Mg (site M5) and Mn (sites M2, M3 and M5) were extremely enriched during the hot-wet season (Figure S2c). ANOVA results showed K, Ca and Mn to be significantly different (*p* < 0.05) across study sites, whereas, all metal variables were significantly different (*p* < 0.001) across seasons, with the exception of Mn (*p* = 0.12) (Table S2).

Regarding sediment Igeo indices, Mg, Cu, Zn and B concentrations indicated no contamination across all seasons (Figure S2d–f), with the exception of cool-dry and hot-wet seasons sites M3 (metal B) and M5 (metal Mg), respectively, which were uncontaminated to moderately contaminated (Figure S2d,f). Sediment Na, K, Ca, Mn and Fe were mostly extremely contaminated, with Fe (all sites, hot-dry and hot-wet season), K (sites M2–M5, cool-dry season), Mn (site M5, cool-dry season), Mn (site M5, cool-dry season; sites M1, M4 and M5, hot-dry season; sites M1, M3 and M4, hot-wet season) being heavily to extremely contaminated (Figure S2e–f). Two-way ANOVA highlighted that Ca, Zn and Mn Igeo index values were significantly different (*p* < 0.05) across study sites, with all study metals Igeo indices being significantly different (*p* < 0.05) across seasons (Table S2).

**Figure 1:**
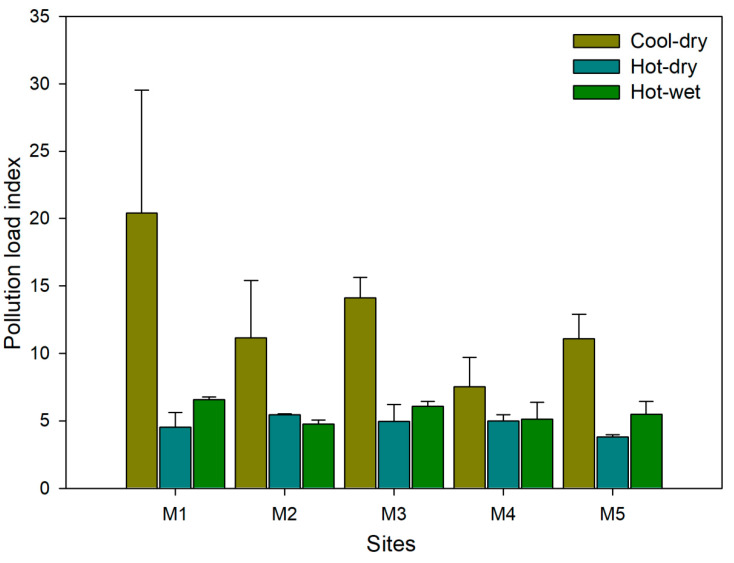
Mean Pollution load indices (±standard error) recorded across five sites (M1–M5) and three seasons (cool-dry, hot-dry, hot-wet) based sediment metal concentrations for the Mvudi River, South Africa.

The sediment metal PLI values indicated deterioration of sediment quality, with high PLI values being observed during the cool-dry season (mean range 7.5 (site M4) to 20.4 (site M1); Figure 1). Low PLI values (mean range 3.8 (site M5) to 5.5 (site M2)) were exhibited during the hot-dry season. The PLI values were found to be significantly different across sites (ANOVA, F_4, 23_ = 1.28, *p* = 0.31) and seasons (ANOVA, F_2, 23_ = 13.16, *p* < 0.001). The cool-dry season had significantly greater PLI than either hot-wet or hot-dry seasons (Tukey HSD: both *p* ≤ 0.001), whilst the hot-dry and hot-wet seasons were statistically similar (Tukey HSD, *p* > 0.05).

### 2.4. Relationship between Sediment Nutrient and Metal Variables

**Figure 2:**
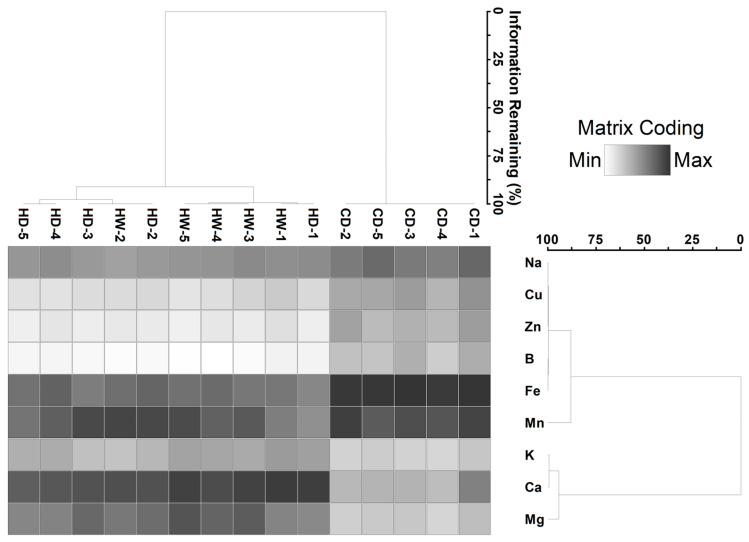
Two-way cluster analysis for sediment chemistry variables across the 5 study sites (i.e., M1–M5) and three seasons (i.e., cool-dry (CD), hot-dry (HD), hot-wet (HW)) from the Mvudi River system, South Africa.

Using PCA analysis, axes 1 and 2 percentage variances were 69.1% (Eigenvalue 6.22) and 14.5% (Eigenvalue 1.30), respectively (Table 2). Factor loadings identified two groupings that consisted of all metals with the exception of Mn, which formed a separate group. The two-way cluster analysis identified two groups; group 1 consisted of Na, Cu, Zn, B, Fe and Mn, and group 2 consisted of K, Ca and Mg (Figure 2). Site and seasonal differences also formed two groupings, with group 1 comprising only cool-dry seasons samples and group 2 consisting of both hot-dry and hot-wet season sites. Group 2 had two subgroups, with subgroup A consisting of site subsets of hot-dry (sites M2–M5) and hot-wet (site M2) seasons, and subgroup B consisting of the other hot-wet (sites M1, M3–M5) and hot-dry (site M1) season sites (Figure 2).

### 2.5. Macrophytes

The roots of *P. australis* had generally high concentrations of Mn, Fe, Cu, Zn and B across all the three seasons, with N, P, Ca and Mg being high in the leaves and K in the stems (Table 3). Sodium was generally similar between root and stem sections. No clearly defined patterns were observed across the study sites and seasons for *P. australis* nutrient and metal concentrations. Three-way ANOVA analysis identified significant differences (*p* < 0.05) for nutrients and metals, with N, Ca, Mg, Na, Zn and B being significant across sites and Ca, Na, Cu, Zn and B being significant across seasons (Table S2). All nutrients and metals differed significantly (ANOVA, *p* < 0.05) across the different *P. australis* plant parts (Table S2).

### 2.6. Bio-Concentration and Translocation of Metals in *Phragmites Australis*

**Figure 3:**
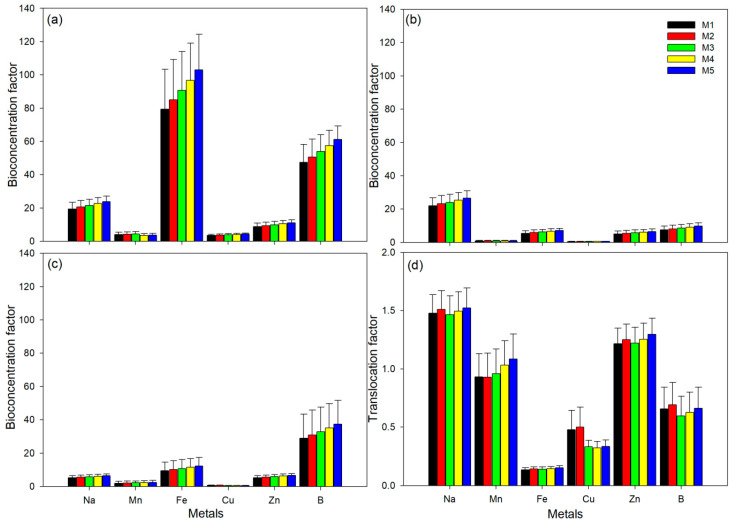
Mean (± standard error) site (M1–M5) variation of bioconcentration (BCF) and translocation (TF) factors of *Phragmites australis* sampled from Mvudi River, South Africa: (**a**) root BCF, (**b**) stem BCF, (**c**) leaves BCF, and (**d**) TF.

The BCF values for *P. australis* were generally high in the roots for all metals with the exception of Na which was high in the stems (Figure 3). Iron and B BCF values exceeded 40 units in the roots (Figure 3a) and B was very high in the leaves, exceeding 25 units across sites. The BCF values increased downstream for all metals across the different plant parts, with site M5 having highest BCF values (Figure 3). The hot-dry and cool-dry seasons had the highest and lowest BCF values, respectively. Significant differences (ANOVA, *p* < 0.05) were observed for Na, Fe, Cu and B BCF values across the different plant parts, Na and Zn BCF values were significantly different (ANOVA, *p* < 0.05) across sites, and BCF values for Na, Fe, Cu, Zn and B were significantly different (ANOVA, *p* < 0.05) among seasons in *P. australis* (Table S2).

The TF values were significantly different (ANOVA, *p* < 0.05) across sites for Fe and B, whereas Fe and Zn TF values were different among the three seasons (ANOVA, *p* < 0.05). TF increased downstream for Na, Mn, Fe and Zn, with that of Cu and B showing a subtly decreasing trend from site M1 to M5 (Figure 3d). The Na TF values were especially high in *P. australis*, ranging between 0.65 (hot-wet season, site M1) and 2.73 (hot-wet season, site M4), whereas TF values in Fe were the lowest and ranged between 0.03 (cool-dry season, site M1) and 0.25 (hot-wet season, site M5).

Using Pearson correlation, similarities (*p* > 0.05) were observed for sediment metals *vs P. australis* roots and leaves metal concentrations (i.e., Na, Mn, Fe, Cu, Zn, B) (Table S3). Significant negative correlations were observed for *P. australis* stem Mn *vs* sediment Mn (*r* = −0.53, *p* = 0.04) and a significant positive relationship between *P. australis* stem Fe *vs* sediment Fe (*r* = 0.54, *p* = 0.04). Root and leaf metal concentrations were always statistically similar (*p* > 0.05). Significant positive correlations were observed for Na (*r* = 0.65, *p* = 0.01), Mn (*r* = 0.59, *p* = 0.02) and Cu (*r* = 0.57, *p* = 0.03) between roots and stems. Furthermore, positive correlations between metal concentration in stems *vs* leaves for Na (*r* = 0.61, *p* = 0.02), Zn (*r* = 0.71, *p* = 0.003) and B (*r* = 0.77, *p* < 0.001) were found.

## 3. Discussion

The present study found that sediment and *P. australis* metal concentrations differed spatiotemporally in the Mvudi River system. Sediment metal concentrations differed across sites and as hypothesised, we observed that superficial sediments had high metal concentrations during the cool-dry compared to hot-wet and hot-dry seasons. Similarly, *P. australis* metal concentrations differed across sites and were higher during the cool-dry season, owing to the seasonality of water flow in the region. *Phragmites australis* metal concentrations were generally higher in the roots than in the stems and leaves, with the exception of Ca and Mg which were higher in the leaves. Relationships between sediment and plant metal concentrations were similarly variable, with only Fe and Mn showing a positive and negative correlation with *P. australis* stems, respectively. Generally, the metal concentrations measured in the present study were in the range reported for other surface sediments in the region [13,20,33,34].

In contrast to the current study, Edokpayi et al. [35] recorded high levels of Cu, Zn, Mn and Fe concentrations during the hot-wet season in Mvudi River. This could be attributed to below average rainfall observed during the hot-wet season during that study. Moreover, some of the metal concentrations recorded in the current study were lower (i.e., Cu, Zn) and others higher (i.e., Fe) than those reported by Dube et al. [21], demonstrating further spatiotemporal variability. Wetland sites M1 and M2 recorded high metal concentrations, and this could be attributed to (i) wetlands generally acting as sinks for nutrients and metals [36], (ii) the proximity of a dumpsite and the use by heavy vehicles visiting the site regularly [37], and (iii) the tributary above site M2 that contained sewage and that drained domestic gardens found in the floodplains [20,38]. Whilst the site (M4) below the sewage treatment works did not have high metal concentrations, a site further downstream i.e., site M5 had greater metal concentrations. These differences could be attributed to sudden increases in water flow from the sewage treatment works which affected metal deposition, occurring further downstream as the channel widened and water flow decreased. More generally, Rajan et al. [6] and Dalu et al. [39] highlighted that during low discharge periods, sediment metals tend to accumulate in freshwater ecosystems and this might have significant effects on the metal bioaccumulation and translocations in flora and fauna.

In addition to hydrological factors, the particle size and components of sediments affect metal concentrations. In particular, finer particles tend to be metal adsorbent, and this tends to affect the physical transportation of metals [22]. Fine particles are also always associated with organic matter, as observed in the current study, and this resulted in metal deposition, especially within the wetland sites from which metals were slowly being released downstream [40,41,42,43]. Yu et al. [7] highlighted that the organic matter influences the metal soil-plant interactions. Analysis of the sediment quality indices indicated that the focal system was of poor health standing generally. Pollution load indices indicated that the system was in stress, highlighting deterioration in sediment quality across the study sites. Most of the metals were, however, found to have originated from a similar source with only K, Ca and Mg originating from sources inconsistent with others. These metals could have been introduced due to one or more anthropogenic activities within the river catchment.

The measured metal concentrations in *P. australis* were higher than those presented in previous literature [17,44]. According to Markert [45], the normal metal concentration in plants is 10 mg kg^−1^ for Cu, 150 mg kg^−1^ for Fe, and 200 mg kg^−1^ for Mn, however, in our study, the metal concentrations often exceeded these values, suggesting that *P. australis* accumulated these metals in marked levels, and particularly in the cool-dry season. Furthermore, concentrations differed between plant parts, whereby roots were most contaminated across most metals. In most sites, *P. australis* plant parts recorded high concentrations of all metals, with the exception of Cu and Mn concentrations compared to the plants’ respective environments. The difference could be attributed to hydrological processes that continuously wash away the metals in river sediment while the *P. australis* continuously bio-concentrate them in the different plant parts. Thus, for the most part, metal uptake into plant tissue is known to differ among plant parts and is further mediated spatiotemporally across metal types. We suspect bio-concentrations were thus affected by metal bioavailability in the soil, rate of absorption by the *P. australis* roots, and translocation from the roots.

The observed metal concentrations for Mn, Fe and Cu exceeded the phytotoxicity limits, i.e., low to severe effect levels based on sediment guidelines [46]. Persaud et al. [46] highlighted that the Cu concentration in sediments should have a lowest effect level (LEL) of 16 mg kg^−1^ to be considered as a cause of biological stress to plants and animals, whereas for Fe the LEL was 20,000 mg kg^−1^ and for Mn 460 mg kg^−1^, with the Mn severe effect level (SEL) being 1100 mg kg^−1^. All *P. australis* plant roots exceeded the LEL for Cu and Fe concentrations across all seasons, with Mn having exceeded SEL in roots, and LEL in stems and leaves. This suggests that these metals might have a negative effect on the general health of *P. australis* and its physiological needs.

Several studies [17,44] have shown *P. australis* to be a good metal accumulator. The present study corroborates those studies, with this species found to have a high phytoaccumulation capability based on their standards, as the BCF was consistently greater than 1 (see Figure 3a–c). However, according to Zhu et al. [47] a good metal accumulator must have the ability of accumulating >5000 mg kg^−1^ metal concentration and bio-concentrating metals with a BCF value of >1000. Hence, from our study, none of these conditions were met as our concentrations and BCF values were lower than the Zhu et al. [47] threshold values, implying that *P. australis* did not play a major role as a metal accumulator.

The high metal concentrations observed in roots suggest some level of metal tolerance through the existence of protective mechanisms limiting these toxic compounds from translocating from roots to stems and leaves [48]. The high metal concentration found in the roots could mainly be attributed to the fact that absorption occurs through the roots. Furthermore, plant physiology also plays an important role in excluding certain metals that are not required by the plant itself, thereby serving to protect the plant above ground components [49]. The distribution of Fe and other metals in *P. australis* consistently followed ordering of roots > stems > leaves. In particular, Fe, B and Cu showed low mobility in regard to metal transport from the roots to leaves, as indicated by the low TF values of <1 (see Figure 3d). This suggests that the sediment type/properties of the study sites could have played a significant role in metal transport. Conversely, *P. australis* had a high translocation capability for Na, Mn and Zn as the TF was > 1, similar to studies by Sochacki et al. [17], Ali et al. [44] and Vymazal et al. [50]. The high and significant metal correlations recorded for different plant parts could suggest that these elements (i.e., Na, Mn and Zn) were transported from the roots to leaves of *P. australis* for growth, where they in turn accumulated. The uptake of these metals is generally high when water pH is <7 as observed in the current study. This is in agreement with Dube et al. [21], Obarska-Pempkowiak et al. [51] and Prajapati et al. [52], who observed that these elements are taken by roots and translocated to the stems and leaves. Furthermore, the strong positive correlation in Fe concentration between sediment and *P. australis* stems indicates that Fe metal uptake is dependent on river sediment concentrations. Contrastingly, the negative significant correlation in the Mn concentration between sediment and *P. australis* stems may suggest that Mn translocation was independent and/or partly inhibited by increased Mn concentration, or by other metals with the sediments.

## 4. Materials and Methods

### 4.1. Study Area

**Figure 4:**
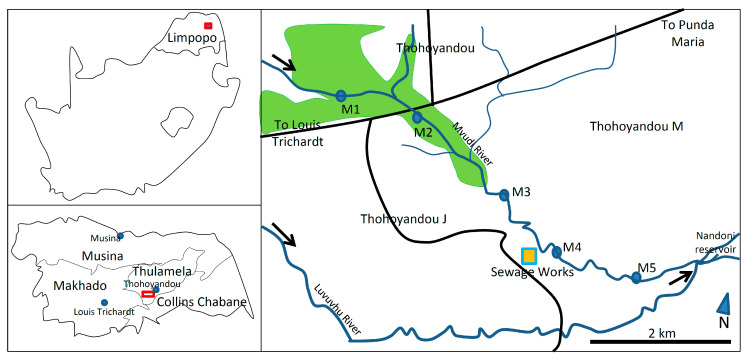
The sampling sites (M1–M5) along the Mvudi River system, Limpopo province, South Africa. Green shading represents wetland areas within the river stretch and black arrows represent direction of river flow.

The study was conducted in Mvudi River, a perennial river and a tributary to the Luvuvhu River system in the Limpopo province of South Africa (Figure 4). The humid, subtropical climate of the region receives an average annual rainfall range of between 400 mm and 800 mm, with peak rainfall occurring between January and February. High temperatures (i.e., up to 40 °C) occur between October and March, with the cool-dry season temperatures ranging between 12 °C and 22 °C. The area soil type is loam which is red in colour due to the presence of iron oxide [53]. This iron oxide in the soil is a result of iron containing ultra-mafic and mafic parent rock, which was formed through physical and chemical weathering [53]. The river system catchment is characterised by agricultural activities, as well as water abstraction, car washing and brick making activities along the riverbanks. Domestic wastewater discharge and spillages from burst pipes from Thohoyandou town are a common occurrence.

Sampling was carried out across the three seasons i.e., cool-dry (June 2019), hot-dry (September 2019) and hot-wet (February 2020) from 5 sites along the Mvudi River (Figure 4). Site M1 was located in a wetland, adjacent to a waste disposal site. Construction vehicles regularly used this site to collect water. Site M2 was also located in a wetland, where there were vegetable gardens along the banks, situated downstream of a tributary that drains a section of Thohoyandou town. Site M3 was located next to an indigenous brick construction company on the riverbank, and also downstream of a major tributary draining the main town centre (i.e., Thohoyandou) and residential areas (Figure 4). Site M4 was <50 m downstream of the Thohoyandou wastewater treatment (TWT) plant discharge point, and site M5 was close to the main reservoir mouth, further downstream of the TWT (Figure 4).

Basic water parameters; conductivity (µS cm^−1^), pH, water temperature (°C) and total dissolved solids (TDS; ppm) were measured using a portable handheld multi–parameter probe (PCTestr 35, Eutech/Oakton Instruments), to better understand the metal accumulation in the plants.

### 4.2. Sediments

Integrated sediment samples (1.5 kg, *n* = 2) were collected from each site and season using a plastic shovel. Sediment from the littoral zones on both banks and middle of the river channel (500 g each), from a depth of 5–10 cm and area of approximately 20 cm × 20 cm were collected and integrated. This was repeated twice (*n* = 2) at each site. Sediment samples were collected by the same person to ensure consistency. The samples were placed in clean polyethylene ziplock bags for transportation to the University of Venda, Department of Ecology and Resource Management Pollution laboratory, Thohoyandou for further processing. In the laboratory, the samples were oven dried at a temperature of 70 °C for 48–72 h. After drying, the samples were ground into powder using a pestle and mortar, thereafter large debris and stones were removed by sieving the samples through a 500 µm mesh size sieve. Further sieving through a 125 µm was conducted to completely remove any remaining plant material, debris and stones.

Detailed methods for cation, metal and nutrients determination are described in Dalu et al. [54]. Briefly, cation elements (i.e., B, Ca, K, Na, Mg) and metals (i.e., Cu, Fe, Mn, Zn) were processed using an ICP-OES optical emission spectrometer (Varian, Mulgrave, Australia), see Dalu et al. [54] for detailed methodology, while total nitrogen and phosphorus were analysed using a SEAL Auto-Analyser 3 and Bray-2 extract as described by Bray and Kurtz [55] and AgriLASA [56] for each site and season. Then, to estimate the accuracy of these methods, a natural standard-certified reference soil, namely SARM-51 (MINTEK) and SL-1 (IAEA), digested and analysed in triplicate, was used for recovery tests. The percentage recoveries of the certified values ranged between 89% and 109%. Lastly, total organic carbon (TOC) was determined using a modified Walkley–Black method as described by Chan et al. [57].

### 4.3. Macrophytes

The diversity of macrophytes was relatively low, with up to six species recorded (see Figure S1), and only *P. australis* was found in all the study sites. Hence, this species was selected for macrophyte metal concentration assessments as it was the most representative and comparable across sites. The dominant macrophyte species (i.e., the common reed *P. australis*, *n* = 5 plants randomly picked) at each site and season was collected by uprooting the entire plant using a shovel while ensuring that all roots were preserved. The collected macrophytes were washed thoroughly to remove any attached sediments and epiphyton. The macrophytes were then cut into three sections (i.e., roots, stem, leaves) and then placed into three separately-labelled polyethylene ziplock bags. The young (sprout) and old (in a state of withering) plant parts were discarded because young plants allocate most of their resources to growth, whereas old plants accumulate chemical metabolites [31]. In addition, macrophyte species cover was visually estimated and expressed as percentage (%) for each site, following Dalu et al. [28] and Hering et al. [58].

After sampling, the leaf blades, roots and stems were washed with a Teepol solution (United Scientific, Johannesburg), rinsed with deionised water and oven dried overnight at 70 °C. The dried leaves were then milled to approximately 40 µm in size and ashed at 480 °C in a furnace, before being mixed with a 50:50 HCl (32 %) solution for extraction through filter paper [59]. The cation and micronutrients (P, K, Ca, Mg, B, Fe, Zn, Cu, Mn) content of each species part extract were measured with a Varian ICP-OES optical emission spectrometer against suitable standards. Total N content of the ground leaves was determined through total combustion in a Leco N-analyser.

### 4.4. Data Analysis

Analysis of variance (ANOVA) models were used to examine the effect of site (5 levels: M1–M5) and season (3 levels: cool-dry, hot-dry, hot-wet) on individual water, sediment and plant parameters. For models considering plant parameters, plant location (3 levels: leaves, stems, roots) was also included, excepting models considering translocation factor. Tukey HSD tests were used for multiple pairwise comparisons, where appropriate. Diagnostic plots confirmed that residuals did not violate parametric test assumptions (i.e., residual normality and homoscedasticity). 

### 4.5. Pollution Indices

#### 4.5.1. Sediment

To determine sediments contamination level by metal at each site and season, the geo-accumulation index was computed. The geo-accumulation index values for different metals were calculated according to Muller [60]:

(1) $$\mathrm{Igeo}={log}_{2}\left(\frac{C_{n}}{1.5B_{n}}\right)$$

where *C_n_* is the concentration of the metal in sediments and *B_n_* is the background of the metal. Seven geo-accumulation indices groups were used to determine the sediment contamination by metals: uncontaminated (Igeo < 0), uncontaminated to moderately contaminated (0 < Igeo < 1), moderately contaminated (1 < Igeo < 2), moderately to heavily contaminated (2 < Igeo < 3), heavily contaminated (3 < Igeo < 4), heavily to extremely contaminated (4 < Igeo < 5) and extremely contaminated (Igeo > 6).

Enrichment factors (EF) were used to assess the contamination of metals in sediment, and this was computed following Buat-Menard and Chesselet [61]:

(2) $$\mathrm{EF}=\frac{{\left(\frac{C_{x}}{Fe_{ref}}\right)}_{sample}}{{\left(\frac{B_{x}}{Fe_{ref}}\right)}_{background}}$$

where *C_x_* is the concentration of the examined metal in a given examined site, *Fe_ref_* is the concentration of the examined Fe metal in the reference site, and *B_x_* is the concentration of the reference metal in a given examined site. The Fe concentration was used as a conservative element to differentiate natural from anthropogenic components within the study, following Li et al. [62]. The enrichment factor categories are as follows: deficiency to minimal enrichment was indicated by EF < 2, moderate enrichment by EF 2–5, significant enrichment by EF 5–20, very high enrichment by EF 20–40 and extremely high enrichment for EF > 40.

Then, to measure the total contamination at each sampling point, the Pollution load index (PLI) was calculated [63]:

(3) $$PLI={\left(C_{f1}\times C_{f2}\times C_{f3}\times \ldots \times C_{fn}\right)}^{1/n}$$

where *n* is number of metals and *C_f_* is the contamination factor. The PLI of each metal is classified as no pollution/perfection (*PLI* ≤ 1), background level pollution (*PLI* = 1) and deterioration of sediment quality (*PLI* > 1).

#### 4.5.2. Macrophytes

To determine the plant’s ability to accumulate metals in respect to the concentration in the sediments, the bio-concentration (BCF) and translocation (TF) factors were calculated and used as indicators [64]. BCF was calculated for the following metals: Na, Mn, Fe, Cu, Zn and B, which had concentrations measured in mg kg^−1^:

(4) $$\mathrm{BCF}=\frac{\left[metal_{plant}\right]}{\left[metal_{sediment}\right]}$$

where *metal_plant_* is the mean concentration in plant biomass and *metal_sediment_* is the metal concentration in sediments [64]. A larger BCF value implies (>1) better phytoaccumulation capability i.e., accumulators and a BCF value < 1 is an excluder.

The TF was used to measure the ability to transfer metals from the roots to the shoots and it is the ratio of metal concentration in the shoots to that of the roots [65]:

(5) $$TF=\frac{C_{s}}{C_{r}}$$

where *C_s_* is the sum of metal concentration in stems and leaves, and the *C_r_* is the metal concentration in the roots. A larger *TF* (>1) value indicates a high translocation capability.

Principal component analysis (PCA) with varimax rotation method was used to determine the natural and anthropogenic sources of sediment metals across study sites (M1–M5) and seasons (cool-dry, hot-dry, hot-wet) in PC-ORD version 5.10 [66]. A two-way cluster analysis, using Ward’s average group linkage method and correlation as a distance measure, was used for metal source identification for the different study sites and seasons. A Pearson correlation was used to test for relationships between sediment metals and plant metals (i.e., leaves, stems, roots) using SPSS version 16 [67].

## 5. Conclusions

The present study found differential pollution indices spatiotemporally in the study system, with significant enrichment found for numerous metals among site and seasons. When considered holistically, pollution loads were always significantly highest during the cool-dry compared to hot-dry and hot-wet seasons, irrespective of sampling site. This thus partly supports our hypothesis as sediments accrued pollutants during the cool-dry, but not hot-dry, periods when water flow is reduced. In turn, the focal macrophyte, *Phragmites australis*, can be considered not to be a major accumulator of metals, but is playing an important role in regulating metals within this study system. Plant metal concentrations were generally most pronounced in the root systems as compared to stems and leaves across metal types. These results provide baseline information for general management of the Mvudi River system and advocates for further investigations into the long-term river water, sediment and plant metal variability, so as to better understand the metal and nutrient dynamics at the water, sediment and plant interface involving several plant species.

## Figures and Tables

**Table 1:** Variation in sediment quality variables recorded across five sites (M1–M5) and three seasons (cool-dry, hot-dry, hot-wet) in the Mvudi River, South Africa.

Sites	P	Na	K	Ca	Mg	Cu	Zn	Mn	B	Fe	S	TOC
	mg kg^−1^	mg kg^−1^	mg kg^−1^	mg kg^−1^	mg kg^−1^	mg kg^−1^	mg kg^−1^	mg kg^−1^	mg kg^−1^	mg kg^−1^	mg kg^−1^	%
*Cool-dry*												
M1	0.03 ± 0.01	240.7 ± 0.3	17.6 ± 5.9	155 ± 33	21.6 ± 8.4	76.6 ± 57.7	46.4 ± 17.9	1307.9 ± 997.8	35.1 ± 21.7	38,894 ± 21,643	22.9 ± 0.02	7.1 ± 1.6
M2	0.02 ± 0.02	179.2 ± 9.7	9.8 ± 0.1	27 ± 1	10.2 ± 1.8	39.2 ± 14.8	42.5 ± 2.8	1858.7 ± 541.1	19.7 ± 21.7	28,333 ± 35,721	5.5 ± 3.7	9.9 ± 2.5
M3	0.03 ± 0.01	187.8 ± 31.2	9.8 ± 2.0	33 ± 1	15.6 ± 2.4	50.3 ± 3.9	33.9 ± 4.6	743.6 ± 199.3	34.6 ± 21.1	55,361 ± 17,421	11.1 ± 1.7	8.5 ± 0.8
M4	0.01 ± 0.01	159.2 ± 8.3	7.8 ± 0.1	23 ± 1	9.0 ± 0.6	29.6 ± 2.3	25.8 ± 0.1	582.1 ± 44.6	10.4 ± 8.3	16,269 ± 15,009	7.3 ± 1.6	6.8 ± 0.9
M5	0.02 ± 0.01	239.1 ± 53	11.7 ± 3.9	30 ± 12	12.6 ± 5.4	39.2 ± 8.2	24.5 ± 2.9	459.8 ± 8.0	18.5 ± 2.8	30,723 ± 4956	14.3 ± 2.8	9.4 ± 3.8
*Hot-dry*												
M1	10.4 ± 2.4	87.4 ± 11.5	42.9 ± 23.4	3780 ± 758	97.8 ± 39.0	6.3 ± 3.4	2.5 ± 0.4	80.6 ± 19.0	0.5 ± 0.1	124.6 ± 50.8	126.4 ± 90.6	4.1 ± 2.8
M2	10.2 ± 3.0	62.1 ± 4.6	27.3 ± 0.1	711.0 ± 29.0	215.4 ± 15	7.0 ± 0.2	2.8 ± 0.1	1140.1 ± 79.2	0.3 ± 0.01	247.3 ± 18.1	14.2 ± 2.5	5.3 ± 1.7
M3	17.1 ± 6.2	62.1 ± 11.5	21.5 ± 5.9	704 ± 166	240.6 ± 60.6	5.8 ± 2.1	2.6 ± 0.5	972.8 ± 477.8	0.3 ± 0.04	176.5 ± 17.9	15.5 ± 1.6	9.0 ± 2.0
M4	50.6 ± 1.2	86.3 ± 10.4	37.1 ± 2.0	470 ± 82.0	147 ± 31.8	4.0 ± 0.6	3.6 ± 0.1	417.5 ± 36.2	0.4 ± 0.02	367.6 ± 9.6	23.4 ± 2.3	5.6 ± 1.0
M5	22.4 ± 1.6	67.9 ± 3.5	35.1 ± 0.1	420 ± 8.0	137.4 ± 0.6	4.1 ± 0.5	2.1 ± 0.3	200.0 ± 15.2	0.3 ± 0.04	209.8 ± 14.7	18.3 ± 4.0	4.9 ± 1.1
*Hot-wet*												
M1	9.0 ± 2.5	81.7 ±5.8	56.5 ± 0.5	4260 ± 20.0	138 ± 6.0	12.1 ± 2.3	4.7 ± 1.8	159.5 ± 0.5	0.5 ± 0.01	193 ± 21.0	54.0 ± 2.4	3.7 ± 0.7
M2	7.0 ± 0.8	42.6 ± 1.2	19.4 ± 0.8	740.0 ± 20.0	192 ± 12.0	6.1 ± 1.2	3.0 ± 0.9	1195 ± 35.0	0.2 ± 0.02	214 ± 30.0	6.6 ± 2.0	4.1 ± 0.3
M3	4.3 ± 1.4	93.2 ± 26.5	37.5 ± 0.1	1390 ± 190.0	438 ± 78.0	9.6 ± 2.5	2.8 ± 1.5	462 ± 61.0	0.3 ± 0.01	193 ± 21.0	4.2 ± 1.0	7.3 ± 4.0
M4	13.9 ± 1.4	74.8 ± 17.3	40.7 ± 9.0	900.0 ± 220.0	252 ± 48.0	5.6 ± 2.2	3.2 ± 0.8	386.5 ± 111.5	0.2 ± 0.03	231.5 ± 66.5	11.1 ± 2.7	5.9 ± 2.2
M5	4.6 ± 1.3	70.2 ± 10.4	40.9 ± 15	1610 ± 31.0	654 ± 138.0	4.0 ± 1.9	0.9 ± 0..03	963 ± 187.0	0.2 ± 0.01	213.5 ± 70.5	5.9 ± 3.3	3.5 ± 0.2

**Table 2:** Principle component analysis (PCA) results for metal concentrations from five sites (M1–M5) and three seasons (cool-dry, hot-dry, hot-wet) for Mvudi River, South Africa. Factor loadings > 0.5 are highlighted in bold.

	Axis 1	Axis 2
Eigenvalue	6.22	1.30
Variance (%)	69.13	14.47
Cum variance (%)	69.13	83.60
*Metals*	*Factor loadings*	
Na	**−0.91**	0.26
K	**0.83**	0.39
Ca	**0.62**	0.60
Mg	**0.65**	−0.39
Cu	**−0.94**	0.19
Zn	**−0.97**	0.07
Mn	−0.51	**−0.69**
B	**−0.96**	0.16
Fe	**−0.94**	0.16

**Table 3:** Mean nutrient and metal concentration recorded in different plant parts of *Phragmites australis* sampled from Mvudi River, South Africa.

Season	N	P	K	Ca	Mg	Na	Mn	Fe	Cu	Zn	B
	%	%	%	%	%	mg kg^−1^	mg kg^−1^	mg kg^−1^	mg kg^−1^	mg kg^−1^	mg kg^−1^
*Root*											
Cool-dry	0.9 ± 0.1	0.07 ± 0.01	0.3 ± 0.1	0.21 ± 0.02	0.09 ± 0.01	1020.8 ± 269.4	3763.4 ± 222.6	32,182 ± 6464	39.4 ± 4.4	37.0 ± 4.4	30.8 ± 7.7
Hot-dry	1.0 ± 0.1	0.08 ± 0.01	0.61 ± 0.1	0.28 ± 0.03	0.11 ± 0.01	2062.2 ± 268.9	2193.8 ± 689.7	27,857 ± 6263	32.0 ± 5.6	38.8 ± 5.6	32.4 ± 7.6
Hot-wet	0.7 ± 0.2	0.05 ± 0.01	0.5 ± 0.2	0.12 ± 0.02	0.07 ± 0.01	1563.4 ± 238.5	725.4 ± 259.9	17,352 ± 471.7	21.0 ± 5.2	20.4 ± 5.2	11.1 ± 0.6
*Stem*											
Cool-dry	1.1 ± 0.2	0.06 ± 0.02	1.7 ± 0.4	0.17 ± 0.07	0.07 ± 0.03	1312.6 ± 299.1	583.4 ± 224.0	1589.6 ± 541.7	5.8 ± 0.9	18.4 ± 2.1	3.4 ± 0.8
Hot-dry	1.0 ± 0.2	0.08 ± 0.02	1.7 ± 0.4	0.15 ± 0.11	0.08 ± 0.03	2364.8 ± 266.1	518.2 ± 238.6	1081 ± 511.88	3.8 ± 1.1	15.8 ± 2.5	3.0 ± 1.2
Hot-wet	1.0 ± 0.2	0.11 ± 0.02	1.6 ± 0.3	0.15 ± 0.07	0.08 ± 0.03	1730.0 ± 300.0	431.0 ± 227.1	2470 ± 391.0	4.2 ± 1.0	17.7 ± 1.8	3.4 ± 0.7
*Leaves*											
Cool-dry	2.4 ± 0.2	0.19 ± 0.03	1.8 ± 0.1	0.43 ± 0.06	0.13 ± 0.01	295.4 ± 51.8	612.8 ± 81.3	1336.6 ± 198.3	27.6 ± 1.0	23.0 ± 3.4	12.2 ± 2.3
Hot-dry	2.3 ± 0.3	0.18 ± 0.02	1.5 ± 0.2	0.62 ± 0.06	0.14 ± 0.01	348.6 ± 46.8	852.0 ± 122.3	1477.4 ± 211.6	4.0 ± 23.4	18.0 ± 4.0	16.6 ± 8.3
Hot-wet	2.5 ± 0.2	0.17 ± 0.04	1.5 ± 0.2	0.30 ± 0.07	0.12 ± 0.02	386.8 ± 68.1	305.0 ± 100.3	1147.8 ± 224.6	2.8 ± 1.1	14.4 ± 3.9	5.0 ± 2.5

## Supplementary Materials

The following are available online at https://www.mdpi.com/2223-7747/9/7/846/s1, Figure S1: The mean (± standard error) macrophyte cover (%) observed across the study sites in the Mvudi River, South Africa; Figure S2. Enrichment factors for (a) cool-dry, (b) hot-dry, and (c) hot-wet season, and the geo-accumulation indices for (e) cool-dry, (f) hot-dry, and (g) hot-wet seasons recorded across five sites for the Mvudi River, South Africa. Error bars are ± standard error; Table S1: Basic water parameters measured from Mvudi River across 5 sites (M1–M5) and 3 seasons (cool-dry, hot-dry, hot-wet). Significant values (*p* < 0.05) are emboldened; Table S2. Analysis of variance (ANOVA) results considering sediment, enrichment factor and geo-accumulation index parameters, and plant, bio-concentration factor and translocation factor parameters as a function of location (leaf, root, stem), site (M1–M5) and season (cool-dry, hot-dry, hot-wet). Significant values (*p* < 0.05) are emboldened; Table S3. Pearson correlation results for metal concentrations in sediments *Phragmites australis* parts, and between different parts within *Phragmites australis*. The numbers in parentheses are *p*-values and bold values are significant at *p* < 0.05.
