# Supplementary material for: Quantifying Metal Contamination and Potential Uptake by Phragmites australis Adans. (Poaceae) Along a Subtropical River System
Source: Plants (Basel). 2020 Jul 4;9(7):846. doi: 10.3390/plants9070846 (PMC7412227; doi:10.3390/plants9070846)
Supplement: Supplementary file 1 [file plants-09-00846-s001.pdf]

Supplementary Files

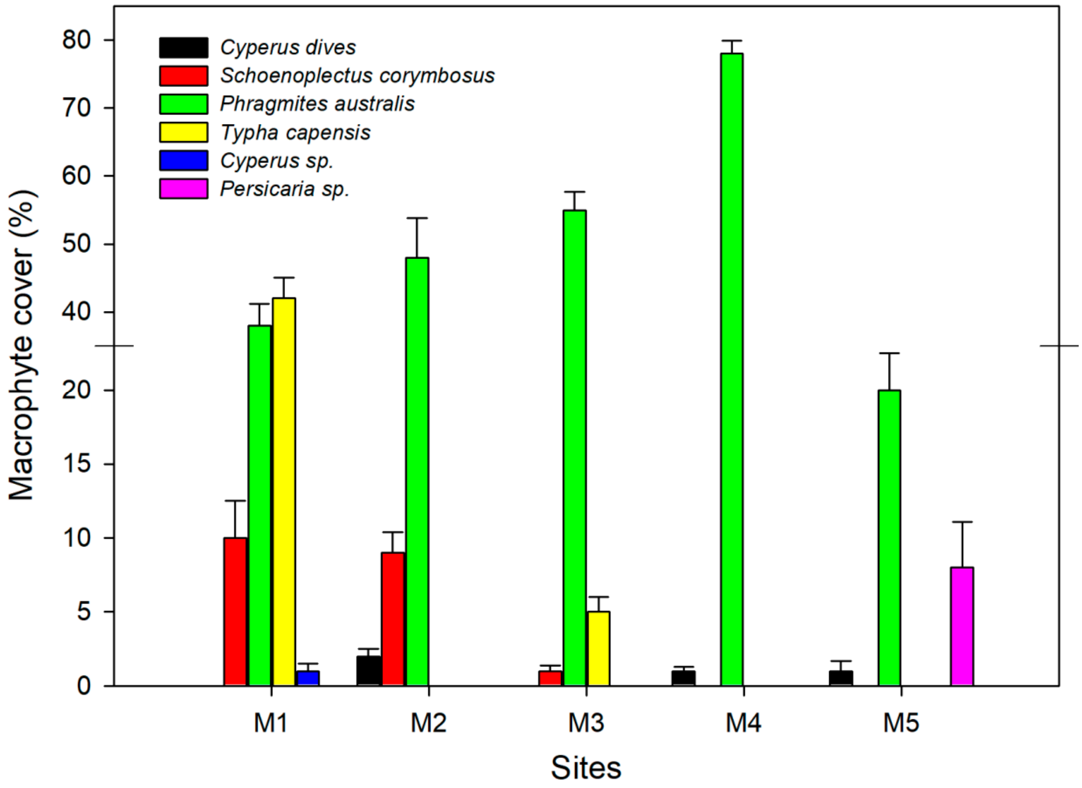

Figure S1. The mean (± standard error) macrophyte cover (%) observed across the study sites in the Mvudi River, South Africa.

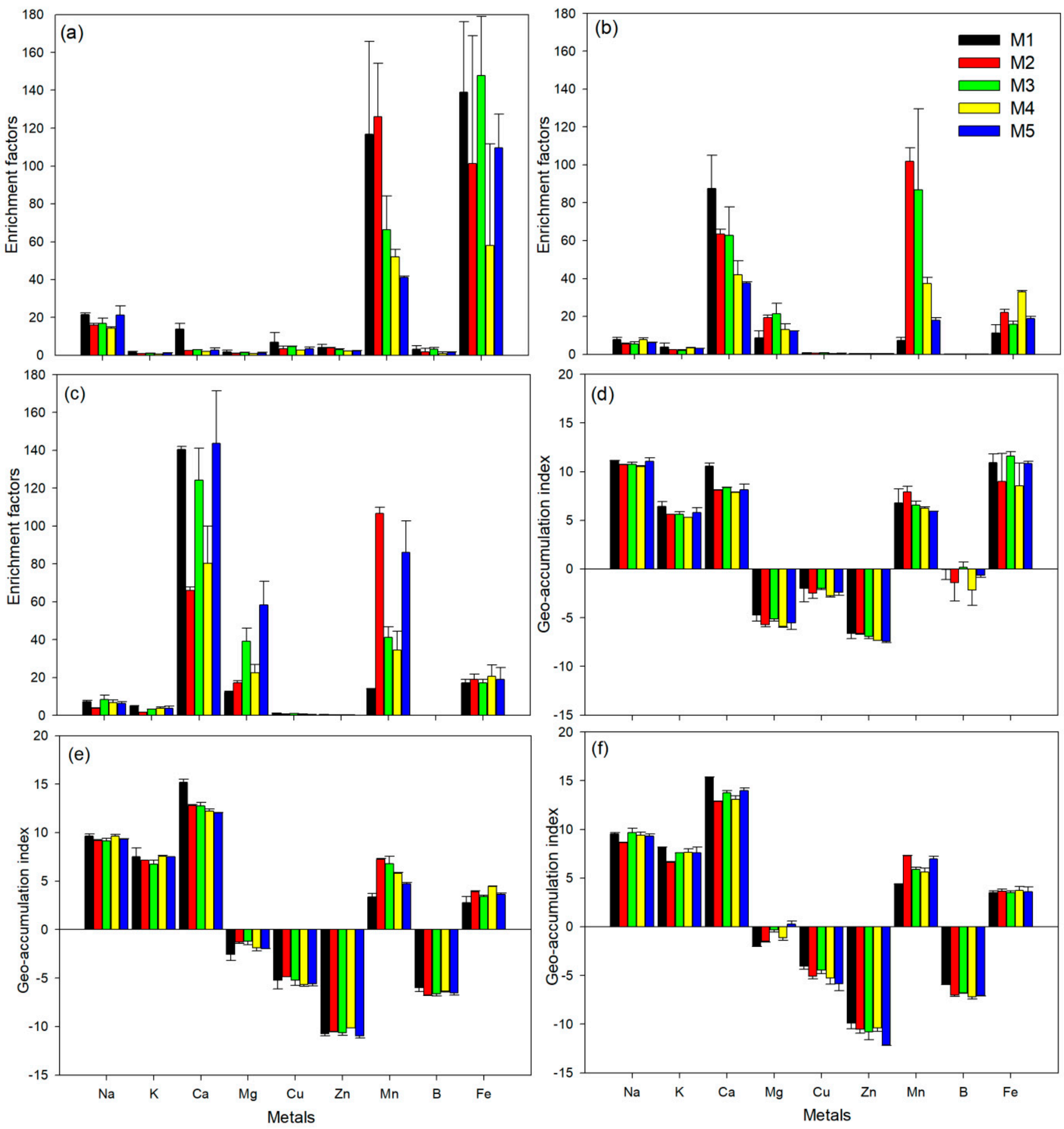

Figure S2. Enrichment factors for (**a**) cool-dry, (**b**) hot-dry, and (**c**) hot-wet season, and the geo-accumulation indices for (**e**) cool-dry, (**f**) hot-dry, and (**g**) hot-wet seasons recorded across five sites for the Mvudi River, South Africa. Error bars are ± standard error.

Table S1. Analysis of variance (ANOVA) results considering sediment, enrichment factor and geo-accumulation index parameters, and plant, bio-concentration factors and translocation factors parameters as a function of location (leaf, root, stem), site (M1–M5) and season (cool-dry, hot-dry, hot-wet). Significant values ($p < 0.05$) are emboldened.

**Parameter**	**Location**		**Site**		**Season**	
	F	***p***	***F* (4, 23)**	***p***	***F* (2, 23)**	***p***
			Sediment			
P	.	.	**4.06**	**0.01**	**19.83**	**< 0.001**
Na	.	.	1.80	0.16	**62.02**	**< 0.001**
K	.	.	**3.11**	**0.04**	**18.74**	**< 0.001**
Ca	.	.	**10.53**	**< 0.001**	**14.61**	**< 0.001**
Mg	.	.	2.48	0.08	**18.65**	**< 0.001**

Cu	.	.	0.84	0.52	**14.38**	**< 0.001**
Zn	.	.	1.49	0.24	**65.19**	**< 0.001**
Mn	.	.	**4.06**	**0.01**	2.38	0.12
B	.	.	0.72	0.59	**16.47**	**< 0.001**
Fe	.	.	0.69	0.61	**18.83**	**< 0.001**
Soluble S	.	.	**3.65**	**0.02**	2.07	0.15
TOC	.	.	0.62	0.75	**7.05**	**0.02**
Enrichment factor						
Na	.	.	1.80	0.16	**62.04**	**< 0.001**
K	.	.	**3.10**	**0.04**	**18.71**	**< 0.001**
Ca	.	.	**10.53**	**< 0.001**	**14.61**	**< 0.001**
Mg	.	.	2.45	0.08	**18.65**	**< 0.001**
Cu	.	.	0.84	0.52	**14.39**	**< 0.001**
Zn	.	.	1.50	0.23	**65.39**	**< 0.001**
Mn	.	.	**4.06**	**0.01**	2.38	0.12
B	.	.	0.72	0.59	**16.46**	**< 0.001**
Fe	.	.	0.69	0.61	**18.83**	**< 0.001**
Geo-accumulation index						
Na	.	.	2.64	0.06	**64.67**	**< 0.001**
K	.	.	2.75	0.05	**35.27**	**< 0.001**
Ca	.	.	**38.57**	**< 0.001**	**425.3**	**< 0.001**
Mg	.	.	1.83	0.16	**122.37**	**< 0.001**
Cu	.	.	1.77	0.17	**52.6**	**< 0.001**
Zn	.	.	**3.69**	**0.02**	**148.2**	**< 0.001**
Mn	.	.	**6.44**	**0.001**	**3.67**	**0.04**
B	.	.	1.86	0.15	**140.48**	**< 0.001**
Fe	.	.	0.25	0.91	**74.23**	**< 0.001**
	**Location DF (2, 36)**		**Site DF (2, 36)**		**Season DF (2, 36)**	
Plants						
N	**33.99**	**< 0.001**	**7.47**	**< 0.001**	0.11	0.9
P	**48.89**	**< 0.001**	0.91	0.47	0.19	0.83
K	**19.34**	**< 0.001**	2.25	0.08	0.11	0.9
Ca	**19.33**	**< 0.001**	**4.43**	**0.01**	**5.00**	**0.01**
Mg	**10.7**	**< 0.001**	**8.65**	**< 0.001**	1.36	0.27
Na	**34.07**	**< 0.001**	**4.77**	**0.003**	**7.18**	**0.002**
Mn	**4.27**	**0.02**	1.48	0.23	1.57	0.22
Fe	**94.46**	**< 0.001**	1.52	0.22	2.77	0.08
Cu	**9.77**	**< 0.001**	2.15	0.09	**3.18**	**0.05**
Zn	**20.25**	**< 0.001**	**4.20**	**0.01**	**6.13**	**0.01**
B	**14.88**	**< 0.001**	**2.85**	**0.04**	**4.22**	**0.02**
Na	**13.67**	**< 0.001**	**5.95**	**< 0.001**	**13.26**	**< 0.001**
Mn	1.91	0.16	0.53	0.71	2.54	0.09
Fe	**9.49**	**< 0.001**	0.9	0.47	**5.30**	**0.01**
Cu	**14.67**	**< 0.001**	1.47	0.23	**6.84**	**0.003**
Zn	2.34	0.11	**6.64**	**< 0.001**	**18.57**	**< 0.001**
B	**4.49**	**0.02**	2.17	0.09	**10.59**	**< 0.001**
Na	.	.	2.13	0.17	0.23	0.98
Mn	.	.	0.95	0.48	0.02	0.98
Fe	.	.	**6.15**	**0.02**	**21.87**	**< 0.001**
Cu	.	.	1.05	0.44	0.85	0.46
Zn	.	.	3.81	0.05	**5.95**	**0.03**
B	.	.	**51.54**	**< 0.001**	1.21	0.35

Table S2. Pearson correlation results for metal concentrations in sediments *Phragmites australis* parts, and between different parts within *Phragmites australis*. The numbers in parentheses are p-values and bold values are significant at $p < 0.05$.

Sediment	Root	Stem	Leaves
Na	-0.39 (0.12)	0.25 (0.37)	-0.28 (0.33)
Mn	0.26 (0.32)	**-0.53 (0.04)**	0.03 (0.93)
Fe	0.22 (0.40)	**0.54 (0.04)**	-0.12 (0.69)
Cu	0.34 (0.19)	-0.22 (0.42)	0.24 (0.40)
Zn	0.27 (0.30)	-0.14 (0.61)	0.25 (0.40)
B	0.29 (0.26)	-0.12 (0.67)	0.04 (0.87)
**Plant**	*leaves vs roots*	*roots vs stems*	*stems vs leaves*
Na	0.51 (0.05)	**0.65 (0.01)**	**0.61 (0.02)**
Mn	-0.13 (0.65)	**0.59 (0.02)**	0.22 (0.43)
Fe	0.12 (0.68)	-0.45 (0.10)	0.19 (0.49)
Cu	0.42 (0.12)	**0.57 (0.03)**	0.42 (0.11)
Zn	0.35 (0.20)	0.20 (0.48)	**0.71 (0.003)**
B	0.13 (0.65)	0.12 (0.67)	**0.77 (< 0.001)**
